# Current drinking and health-risk behaviors among male high school students in central Thailand

**DOI:** 10.1186/1471-2458-11-233

**Published:** 2011-04-14

**Authors:** Wisit Chaveepojnkamjorn, Natchaporn Pichainarong

**Affiliations:** 1 Department of Epidemiology, Faculty of Public Health, Mahidol University, Ratchathewi, Bangkok, Thailand; 2 Faculty of Public Health, Mahasarakham University, Kantharawichai, Maha Sarakham, Thailand

## Abstract

**Background:**

Alcohol drinking is frequently related to behavioral problems, which lead to a number of negative consequences. This study was to evaluate the characteristics of male high school students who drink, the drinking patterns among them, and the associations between current drinking and other health risk behaviors which focused on personal safety, violence-related behaviors, suicide and sexual behaviors.

**Method:**

A cross-sectional study was conducted to explore current alcohol drinking and health-risk behaviors among male high school students in central Thailand. Five thousand one hundred and eighty four male students were classified into 2 groups according to drinking in the previous 30 days (yes = 631, no = 4,553). Data were collected by self-administered, anonymous questionnaire which consisted of 3 parts: socio-demographic factors, health-risk behaviors and alcohol drinking behavior during the past year from December 2007 to February 2008.

**Results:**

The results showed that the percent of current drinking was 12.17. Most of them were 15-17 years (50.21%). Socio-demographic factors such as age, educational level, residence, cohabitants, grade point average (GPA), having a part time job and having family members with alcohol/drug problems were significantly associated with alcohol drinking (p < 0.05). Multiple logistic regression analysis, after adjusting for socio-demographic factors, revealed that health-risk behavioral factors were associated with current alcohol consumption: often drove after drinking alcohol (OR = 3.10, 95% CI = 1.88-5.12), often carried a weapon (OR = 3.51, 95% CI = 2.27-5.42), often got into a physical fight without injury (OR = 3.06, 95% CI = 1.99-4.70), dating violence (OR = 2.58, 95% CI = 1.79-3.71), seriously thought about suicide (OR = 2.07, 95% CI = 1.38-3.11), made a suicide plan (OR = 2.10, 95% CI = 1.43-3.08), ever had sexual intercourse (OR = 5.62, 95% CI = 4.33-7.29), alcohol or drug use before last sexual intercourse (OR = 2.55, 95% CI = 1.44-4.53), and got someone pregnant (OR = 3.99, 95% CI = 1.73-9.25).

**Conclusions:**

An increased risk of health-risk behaviors, including driving vehicles after drinking, violence-related behaviors, sad feelings and attempted suicide, and sexual behaviors was higher among drinking students that led to significant health problems. Effective intervention strategies (such as a campaign mentioning the adverse health effects and social consequences to the risk groups, and encouraging parental and community efforts to prevent drinking) among adolescents should be implemented to prevent underage drinking and adverse consequences.

## Background

It is well established that an increase in alcohol consumption leads to a higher proportion of persons with problem use and addiction. In 2007, the National Statistical Office of Thailand found 29.3% of Thais ≥ 15 years old consume alcohol. The proportion of male alcohol consumption in the age 25-59 years was 59.1%, 15-24 years 39.2% and over 60 years 29.4%, respectively [1]. It was found that the proportion of male drinkers increased from 55.4% in 1996 to 60.8% in 2003, but dropped to 50.3% in 2006 and 51.0% in 2007 [1,2]. During 1996-2006, the proportion of male drinkers has risen in young people, particularly those aged 15-19 years (20.8% to 24.2%) and aged 20-24 years (56.0% to 58.1%) [2]. Reports from the National Statistical Office of Thailand in 2007 showed the major alcoholic risk groups were working people and youths. Alcohol drinking is frequently related to behavioral problems, which lead to a number of negative consequences, such as domestic violence and quarrels with neighbors, suicides and sexual assault and risky sexual behaviors. It also accounts for numerous accidents such as injuries and traffic accidents. Youths who drink have a greater tendency to develop those health-risk behaviors.

The aim of this study was to evaluate the characteristics of male high school students who drink, the drinking patterns among them, and the associations between current drinking and other health risk behaviors which focused on personal safety, violence-related behaviors, suicide and sexual behaviors.

## Methods

### Study design and population

A cross-sectional study was conducted from December 2007 to February 2008 in order to explore current drinking and health-risk behaviors among male high school students in central Thailand. The proposal was reviewed and approved by the Ethics Committee for Research in Human Subjects of Mahidol University (Ref.No. Mu 2007-243). A two-stage stratified sampling technique was used to select the 5,184 students from 10 provinces of central Thailand within the academic zones 5, 6, and 12. The primary sampling unit was province and the second was school. These provinces were randomly selected and represented characteristics of male adolescents in central Thailand. In each province, we randomly selected at least one school from the list of each of the three school categories: urban and rural public schools, and private schools. The selection of schools was based on a list of schools obtained from the Provincial Education Offices and willingness of school administrators to participate in the study. Selected schools could refuse to participate in the study. Altogether, 5,184 students from Mathayomsuksa School (MS) 1, 3 and 5 participated in the study (equivalent to grade 7, 9 and 11 of an ordinary school). In each school, 3 or fewer classes of each of these 3 educational levels were recruited from the same grade level. If there were more than three classes, three classes with students of mixed academic performance were randomly selected by their teachers. The study subjects were classified into 2 groups according to their alcohol intake during the 30 days preceding the survey (yes = 631, no = 4,553). Information was collected by a self administered, anonymous questionnaire which consisted of 3 parts, socio-demographics, health-risk behaviors during past year and alcohol drinking behavior with the help and supervision of well trained research assistants. Current drinking was defined as drinking at least one standard drink of an alcoholic beverage during the past 30 days of the survey. A standard drink was defined as a can (330 cc) of beer, a glass (100 cc) of wine, or a small glass (30 cc) of whisky or spirits. The health-risk behaviors questionnaire was modified from the questionnaire of the U.S. Youth Risk Behavior Surveillance System [3,4] and focused on personal safety, violence-related behaviors, suicide and sexual behaviors. The Thai version was translated and verified by a bilingual English university lecturer, and it was reviewed by the experts (composed of psychiatrists, psychologists, nurse, social workers, school teachers and health promotion specialists). Details of the study were explained and an informed consent form was signed. Individual answers were kept confidentially.

### Statistical analysis

Socio-demographic factors were given as a percentage, crude odds ratio, 95% CI of OR and p-value. Univariate analysis was performed using the Pearson's chi-square test to differentiate proportional exposures between drinkers and non-drinkers for categorical variables. Adjusted odds ratio and the 95% CI of OR were calculated from multiple logistic regression to examine associations between current drinking and health-risk behaviors, adjusted for socio-demographic factors. A criteria of p < 0.05 for statistical significance was applied.

## Results

A total of 5,184 male high school students, their percents of alcohol consumption in the past 30 days were 12.17. Current drinking prevalence increased with increasing age (6.41% among less than 15 year-olds vs 26.54% among 18 years and older) and grade level (4.73% among 7^th ^graders vs 22.08% among 11^th ^graders).

Using a univariate analysis, the factors significantly associated with current alcohol drinking assessed by a Pearson's chi-square test (p < 0.05) were age group (OR 15-17 yrs = 2.92; 95% CI = 2.40-3.53; OR ≥18 yrs = 5.28, 95%CI = 3.59-7.76), educational level (OR MS 3 = 3.06, 95% CI = 2.38-3.95; OR MS 5 = 5.74, 95% CI = 4.46-7.40), residence (OR school dormitory = 1.82, 95% CI = 1.07-3.08), cohabitants (OR friend = 2.93, 95% CI = 1.76-4.87), as well as GPA (OR GPA < 2.0 = 1.36, 95% CI = 1.05-1.76; OR GPA > 3.0 = 0.42; 95% CI = 0.32-0.56), having a part time job (OR = 1.47, 95% CI = 1.22-1.76) and having family members with alcohol/drug problems (OR = 1.26, 95% CI = 1.05-1.51) (Table 1).

**Table 1:** Socio-demographic factors associated with current drinking of male high school students

Variables	No.drinker/total	%drinker	Crude OR	95%CI	**p-value **^**a**^
Age group (yrs) (n = 5184)					
<15	155/2419	6.41	1		
15-17	433/2603	16.63	2.92	2.40-3.53	<0.001
≥ 18	43/162	26.54	5.28	3.59-7.76	<0.001
Educational level (n = 5184)					
MS 1	94/1987	4.73	1		
MS 3	252/1906	13.22	3.06	2.38-3.95	<0.001
MS 5	285/1291	22.08	5.74	4.46-7.40	<0.001
Religion (n = 5169)					
Buddhist	615/5054	12.17	1		
Islam	4/33	12.12	0.98	0.29-2.94	1.000
Others	10/82	12.19	0.99	0.48-1.99	0.973
Residence (n = 4673)					
House/Apartment of family	552/4534	12.18	1		
School dormitory	20/98	20.41	1.82	1.07-3.08	0.017
Private dormitory	7/41	17.07	1.47	0.59-3.47	0.358
Cohabitants(n = 4943)					
Parent	500/4157	12.03	1		
Relative	75/638	11.76	0.96	0.74-1.25	0.761
Friend	24/84	28.57	2.93	1.76-4.87	<0.001
Alone	9/64	14.06	1.20	0.55-2.54	0.612
Grade point average. (n = 4461)					
<2.0	99/535	18.50	1.36	1.05-1.76	0.016
2.0-3.0	307/2125	14.45	1		
>3.0	160/1801	8.88	0.42	0.32-0.56	<0.001
Part time job (n = 5120)					
No	411/3724	11.04	1		
Yes	213/1396	15.26	1.47	1.22-1.76	<0.001
Having family members with alcohol/drug problems (n = 4402)					
No	297/2607	11.39	1		
Yes	249/1795	13.87	1.26	1.05-1.51	0.014

^a ^Pearson's chi-square test, OR = odds ratio, CI = confidence interval

MS 1 = 1^st ^year of secondary school (equivalent to 7^th ^grader), MS 3 = 3^rd ^year of secondary school (equivalent to 9^th ^grader, MS 5 = 5^th ^year of secondary school (equivalent to 11^th ^grader)

Other health risk behaviors were more common among current drinkers. After adjusting for socio-demographic factors (age, educational level, residence, cohabitants, GPA, having a part time job, and having family members with alcohol/drug problems), compared with non-drinkers, current drinkers were more likely to drive a car/bicycle after drinking alcohol (OR = 3.10, 95% CI = 1.88-5.12). For violence-related behaviors, often carrying a weapon (OR = 3.51, 95% CI = 2.27-5.42), often getting into a physical fight without injury (OR = 3.06, 95% CI = 1.99-4.70), and experience dating violence (OR = 2.58, 95% CI = 1.79-3.71) were proportionally higher among current drinking students than non-drinkers. In addition, sad feelings and attempted suicide, indicated that participants had seriously thought about suicide (OR = 2.07, 95% CI = 1.38-3.11) and making a suicide plan (OR = 2.10, 95% CI = 1.43-3.08) were also significantly associated with current drinking. In case of sexual behaviors, higher proportions of ever having sexual intercourse (OR = 5.62, 95% CI = 4.33-7.29), alcohol or drug use before last sexual intercourse (OR = 2.55, 95% CI = 1.44-4.53), and getting someone pregnant (OR = 3.99, 95% CI = 1.73-9.25) were found among current drinkers than among non-drinking students, as shown in Table 2.

**Table 2:** Percent, crude OR and adjusted OR of health-risk behaviors among male high school students according to drinking status

Health-risk behaviors	All Respondent (n = 5184)	Nondrinkers (4553)		Current drinkers (n = 631)				
	**%**	**%**	**Adjusted OR**^**a**^	**%**	**Crude OR**	**Adjusted OR**	**95%CI**	**p-value**

**Personal safety**								
Never or rarely wore bicycle helmets	67.76	67.95	1	66.49	0.93	1.19	0.96-1.48	0.113
Never or rarely wore seat belts	61.84	61.71	1	62.77	1.04	1.18	0.95-1.48	0.138
Often drove a car/motorcycle after drinking alcohol	2.24	1.61	1	6.74	4.43	3.10	1.88-5.12	<0.001
**Violence-related behaviors**								
Often carried a weapon	3.38	2.61	1	8.82	3.61	3.51	2.27-5.42	<0.001
Often been threatened or injured with a weapon	1.39	1.29	1	2.13	1.66	1.14	0.53-2.46	0.737
Often got into a physical fight without injury	3.54	2.72	1	9.31	3.67	3.06	1.99-4.70	<0.001
Often got into a physical fight with serious injury	1.46	1.38	1	1.96	1.43	1.39	0.67-2.91	0.378
Dating violence	10.61	8.71	1	19.06	2.47	2.58	1.79-3.71	<0.001
Ever forced to have sexual intercourse	5.89	4.96	1	9.65	2.05	1.76	0.98-3.15	0.058
**Sad feelings and attempted suicide**								
Seriously thought about suicide	4.02	3.40	1	8.49	2.66	2.07	1.38-3.11	<0.001
Made a suicide plan	4.51	3.80	1	9.62	2.69	2.10	1.43-3.08	<0.001
**Sexual behaviors**								
Ever had sexual intercourse	8.73	5.68	1	30.58	7.32	5.62	4.33-7.29	<0.001
Had ≥ 4 sex partners during lifetime	28.02	24.62	1	33.67	1.56	1.29	0.80-2.09	0.288
Alcohol or drug use before last sexual intercourse	12.80	12.06	1	22.07	2.94	2.55	1.44-4.53	0.001
Condom use during last sexual intercourse	57.14	50.14	1	74.15	2.85	1.48	0.85-2.56	0.164
Birth control use before last sexual intercourse	58.88	52.29	1	72.82	2.44	1.26	0.79-2.02	0.337
Had got someone pregnant	6.58	4.84	1	11.11	2.46	3.99	1.73-9.25	0.001

^a ^Adjusted for age gr., educational level, residence, cohabitants, GPA, part time job and family members with alcohol/drug problems

**Figure 1:**
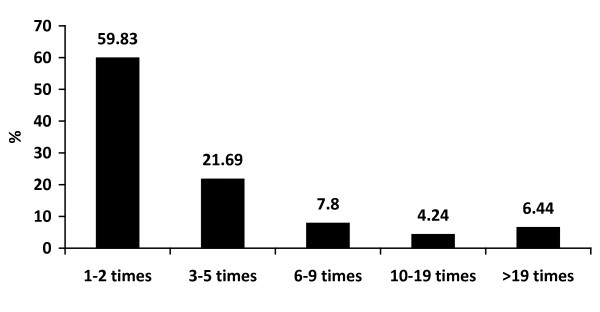
**Frequency of drinking in the previous month**.

**Figure 2:**
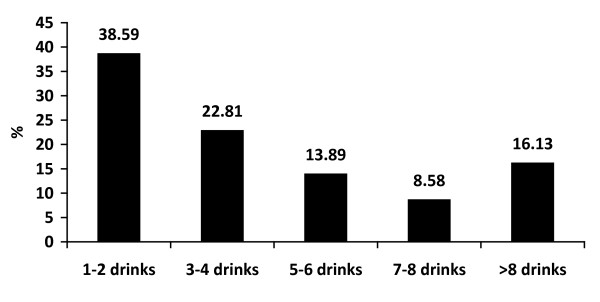
**Quantity of drinking each time in the previous month**.

**Figure 3:**
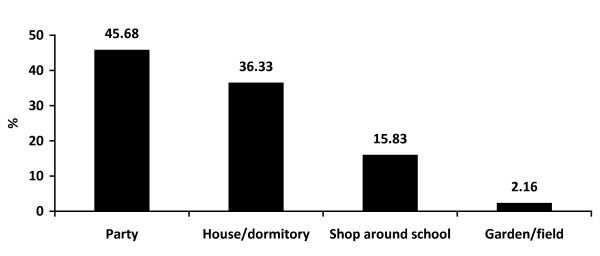
**Places of drinking**.

**Figure 4:**
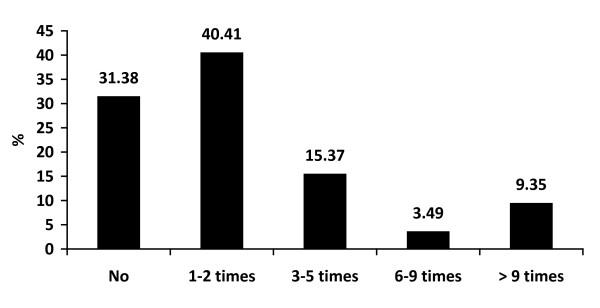
**Frequency of drinking each time ≥5 drinks within 2 weeks**.

**Figure 5:**
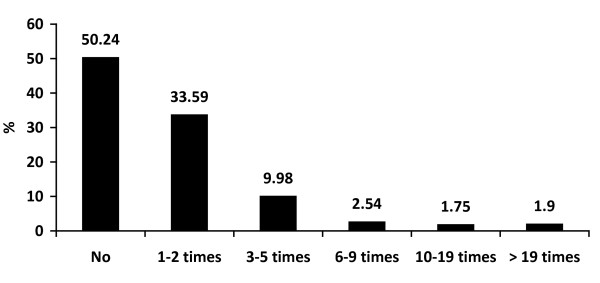
**Frequency of drinking until intoxication in the previous month**.

The majority of boys drank alcohol less than 3 times a month (59.83%) and they each consumed less than 3 standard drinks in the previous month (38.59%). Approximately 40% of boys drank more often than twice a month and 39% of them drank more than 4 standard drinks each time. Alcohol consumption was more common when joining a party (45.68%) but a high proportion of boys drank at home/dormitory (36.33%). Nearly 70% of respondents experienced binge drinking within 2 weeks and nearly half of them admitted to being intoxicated during the 30 days prior to participating in this study, as shown in Figure 1, 2, 3, 4 and 5.

## Discussion

The present study found an increased risk of health-risk behaviors, including intentional injury-related behaviors, suicidal behaviors and premature sexual behaviors among drinking and non-drinking students. This study is one of a few studies on underage drinking among high school students in a developing country of the South-East Asian region. Our findings demonstrated that approximately 12% of male high school students reported that they drank alcohol during the 30 days preceding the survey. This figure was also lower than the studies of Miller *et al*, 2007 [5], Johnston *et al*, 2008 [6] and Assanangkornchai *et al*, 2007 [7]. Even more alarming, we found that among students who drink, almost 70% reported binge drinking (drinking ≥ 5 drinks of alcohol in a row at least once during the past 2 weeks) and half of them had experienced drunkenness. Heavy episode drinking in adolescence is associated with several serious consequences; such as motor vehicle injuries, suicide, sexual assault and risky sexual behaviors with the danger to acquire sexual transmitted diseases and unwanted pregnancies [8]. Adolescence is a vulnerable period and facilitates the start of risk behaviors, for instance consuming alcohol, cigarette smoking and substance use. This study confirmed that older boys drank more than the younger ones [5,9-13]. Non-drinkers had lower rates of health risk behaviors compared with current drinkers. This study also confirmed that current drinking is associated with driving a vehicle which corresponds with the results of the study of Domingues *et al*, 2009 [14]. Eaton found 10% of high school students had driven a car or other vehicle one or more times when they had been drinking alcohol [15]. Alcohol is associated with much of the mortality and morbidity among youth. In addition, alcohol drinking and drunk driving are a major factor causing road traffic accidents. The percentage of Thai male drunk drivers has risen from 36.6 in 2001, to 48.2 in 2002 and 44.1 in 2006 [2]. In aspect of violence-related behaviors, current drinkers are at higher risk of often carrying a weapon [16-18], getting into a physical fight without injury [5] and dating violence [5,19-21]. In addition, according to the present study (see table 2 above) drinkers are at higher risk of seriously thinking about suicide and making a plan how they would attempt suicide. This corresponds with the results of the previous studies [5,22-24]. In sexual behaviors, this study also confirmed that higher levels of alcohol consumption are associated with ever having sexual intercourse [5,20,25], alcohol/drug use before last sexual intercourse [5,26] and getting someone pregnant [5,22,27].

### Limitation of the study

Some limitations of this study should be noted. First, the study was a cross-sectional study, therefore, a temporal relationship cannot be established between alcohol consumption and the health-risk behaviors. A longitudinal study would be needed to examine causal effects of drinking on subsequent drinking. Second, all data were obtained through self-reports, which may lead to inaccuracies of alcohol drinking [28-31] and other health-risk behaviors.

## Conclusions

An increased risk of health-risk behaviors, including driving vehicles after drinking, violence-related behaviors, sad feelings and attempted suicide, and sexual behaviors was higher among drinking students that led to various health problems. The results from this study show prevention of underage drinking is needed. Law enforcement, namely minimum age of purchasing alcohol ≥ 20 years, increasing the penalty of drunk driving, and limiting the availability and accessibility of alcohol through restrictions of times and places should be done strictly. The results suggested that effective intervention strategies among adolescents should be implemented to prevent underage drinking. Applications of the health promoting school model of WHO [32] should be utilized to develop the effective school health programmes to relieve these problems. Some papers showing the effectiveness of Health Promoting School in minimising health-risk behaviors [33-39]. Therefore attempts should be made to limit youth access to alcohol in order to reduce accidents, injuries, violence and alcohol-related health problems [40]. A campaign mentioning the adverse health effects and social consequences to the risk groups, and encouraging parental and community efforts to prevent youth drinking and other health-risk behaviors would reduce the proportion of new and current drinkers and other adverse consequences. The results obtained from this study indicate that to prevent drinking among adolescents need a rather sophisticated approach and more studies to explore the cultural and socio-economic background of current youth drinkers.

## Competing interests

The authors declare that they have no competing interests.

## Authors' contributions

WC was involved in drafting the manuscript and revising it critically for important intellectual content. All authors were involved in the design of the study, statistical analysis and data interpretation. All authors have read and approved the final manuscript.

## Pre-publication history

The pre-publication history for this paper can be accessed here:

http://www.biomedcentral.com/1471-2458/11/233/prepub
